# LC-HRMS Metabolomics for Untargeted Diagnostic Screening in Clinical Laboratories: A Feasibility Study

**DOI:** 10.3390/metabo8020039

**Published:** 2018-06-15

**Authors:** Bertrand Rochat, Rayane Mohamed, Pierre-Edouard Sottas

**Affiliations:** 1 Protein Analysis Facility, Center for Integrative Genomics (CIG), University of Lausanne, CH-1015 Lausanne, Switzerland; 2 Département Formation Recherche, Centre Hospitalier Universitaire Vaudois (CHUV), CH-1011 Lausanne, Switzerland; rayane.mohamed@gmail.com; 3 Biokaizen Lab SA, 1870 Monthey, Switzerland; pesottas@gmail.com

**Keywords:** clinical/biomedical analysis, diagnostic, forensic/toxicology, high resolution mass spectrometry, liquid chromatography, metabolomics, screening, untargeted

## Abstract

Today’s high-resolution mass spectrometers (HRMS) allow bioanalysts to perform untargeted/global determinations that can reveal unexpected compounds or concentrations in a patient’s sample. This could be performed for preliminary diagnosis attempts when usual diagnostic processes and targeted determinations fail. We have evaluated an untargeted diagnostic screening (UDS) procedure. UDS is a metabolome analysis that compares one sample (e.g., a patient) with control samples (a healthy population). Using liquid chromatography (LC)-HRMS full-scan analysis of human serum extracts and unsupervised data treatment, we have compared individual samples that were spiked with one xenobiotic or a higher level of one endogenous compound with control samples. After the use of different filters that drastically reduced the number of metabolites detected, the spiked compound was eventually revealed in each test sample and ranked. The proposed UDS procedure appears feasible and reliable to reveal unexpected xenobiotics (toxicology) or higher concentrations of endogenous metabolites. HRMS-based untargeted approaches could be useful as preliminary diagnostic screening when canonical processes do not reveal disease etiology nor establish a clear diagnosis and could reduce misdiagnosis. On the other hand, the risk of overdiagnosis of this approach should be reduced with mandatory biomedical interpretation of the patient’s UDS results and with confirmatory targeted and quantitative determinations.

## 1. Introduction

Diagnostic process starts from the patient’s symptoms and history. It is evidence-based and tries to rule in or out different disease possibilities and therefore relies on targeted/knowledge-driven approaches. Proper diagnosis usually includes laboratory measurements of known biomarkers, predefined before their determinations, together with biomedical interpretations by various specialists [1,2]. However, diagnosis errors—incomplete, inaccurate, or delayed diagnoses—are real concerns [3,4], even though common biases are known [1,2]. As stated recently by a report of the American Health and Medicine Division, diagnostic errors have been widely undervalued in the delivery of quality health care, continue to harm a significant number of patients and should likely worsen as the delivery of health care is more and more individualized and complex [3].

**Figure 1:**
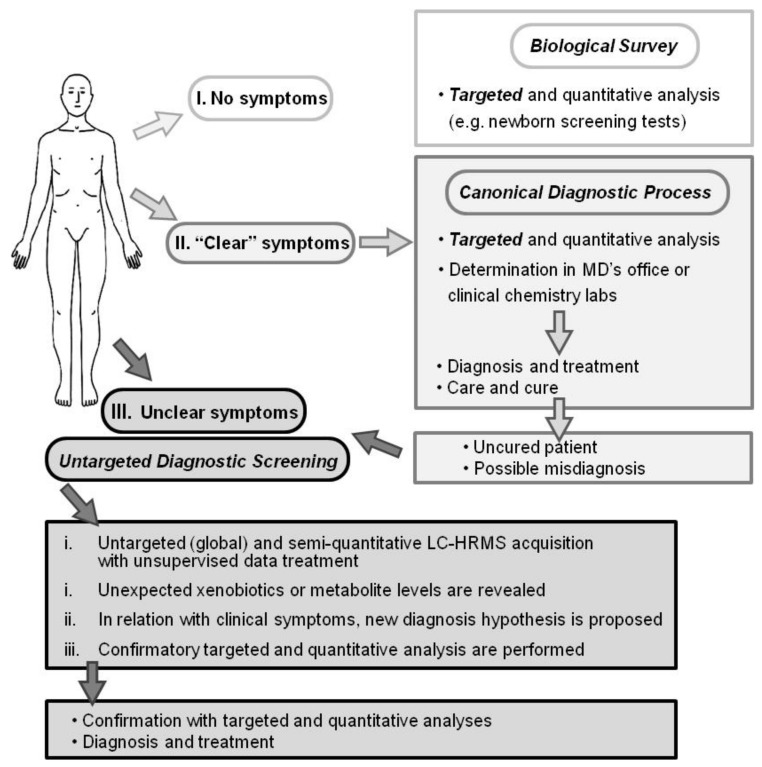
Diagnostic processes depicted as I. a biological survey (e.g., inborn error screening tests), II. Canonical practice (usual symptoms and diseases), and III. Untargeted diagnostic screening (UDS; uncommon symptoms or diseases). Whereas biological survey and canonical diagnostic processes rely on targeted and quantitative analysis, UDS is an untargeted, global, and metabolomic analysis and relies on today’s high-resolution mass spectrometer capabilities. The UDS procedure and data treatment require more than 6 h and possibly further confirmation analyses. UDS cannot be performed for urgent diagnosis.

**Figure 2:**
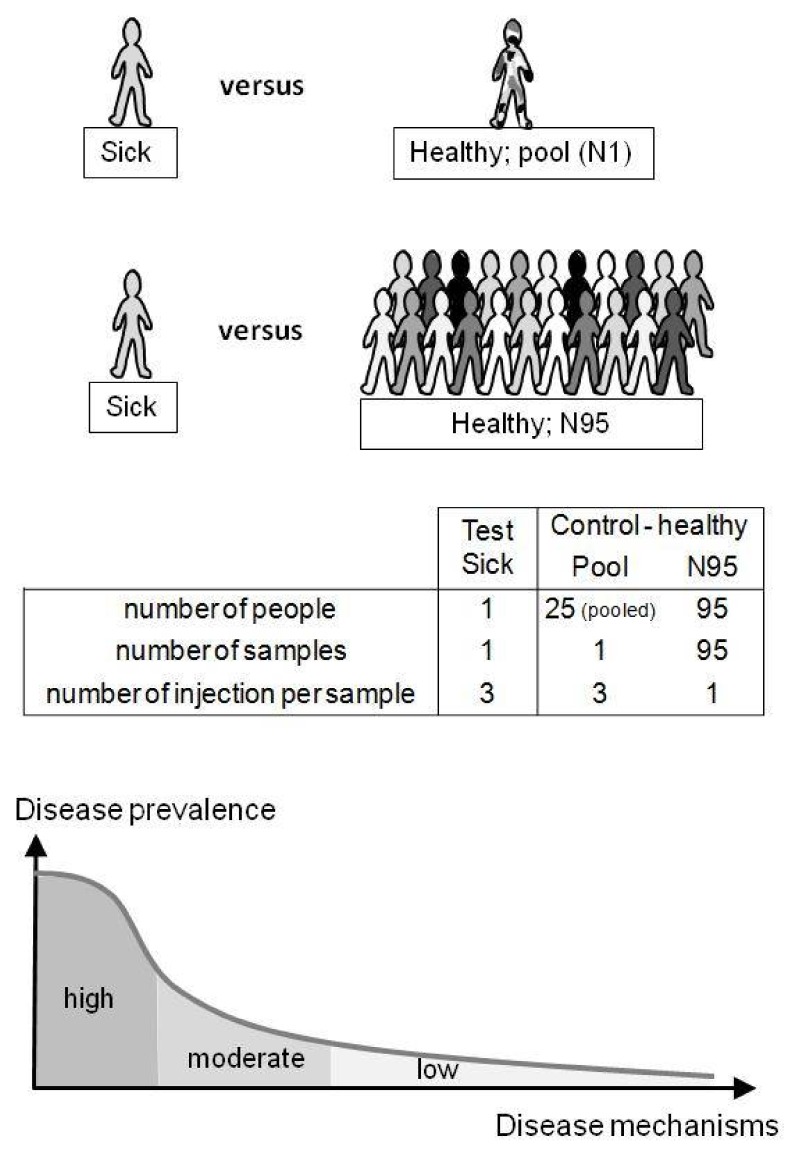
Untargeted diagnostic screening (UDS) compares one person’s metabolome (test) with healthy metabolomes (control). In this study, the control group was a pooled sample (*N* = 1) of identical serum volumes from 25 different healthy donors (Pool) or 95 different serum samples from healthy donors (*N* = 95). The uniqueness of the patient’s metabolome is taken into account (N-of-1 study) and does not necessarily have to belong to a group of patients (“tyranny of the average”). UDS can be of interest when considering misdiagnosis and the high number of low-prevalence diseases (“long-tail disease”; bottom).

Thus, the determination of compounds, known prior to the analysis and representing biomarkers of a suspected health condition, is commonly used in differential diagnostic process [1,2]. However, there are less common diseases and clinical symptoms in which such targeted approaches do not properly reveal disease etiology nor establish a clear diagnosis (Figure 1). Such diseases may benefit from an untargeted diagnostic screening (UDS) approach in which a much larger number of compounds (*N* > 1000; the metabolome) can be measured in one person’s sample whereas no metabolites are predefined before their measurements (Figure 1) [5,6,7,8,9,10,11]. However, in contrast to most determinations of the metabolome, UDS does not discover new biomarkers from a group of sick persons (versus a healthy population) but compares one person’s metabolome with healthy people’s metabolomes (Figure 2). UDS is a metabolomic analysis (see the second following paragraph) with the goal of revealing unexpected metabolites that could lead to new diagnostic hypotheses. Thus, UDS could be seen as a N-of-1 study [12,13], where the patient’s metabolome is seen as unique and does not have to be put in a large group of patients. UDS could also be seen as a fight against the “tyranny of the average”, which lacks consideration of the huge heterogeneity and number of disease origins and mechanisms (low prevalence or rare diseases) [14,15]. Thus, UDS is part of personalized medicine in the sense that, with this unsupervised approach, a patient is seen as a person with unique biological characteristics and not as a member of a group that can be entered into general clinical practice guidelines [16,17]. This is in agreement with the fact that the same symptoms or disease can have a huge number of different mechanisms showing long tail prevalence (Figure 2, bottom). Indeed, as raised by RC Ziegelstein [17], when medicine follows strict clinical practice guidelines, it does not consider patients as unique individuals but as persons that should be part of subgroups and considers individual variations of minor importance.

**Figure 3:**
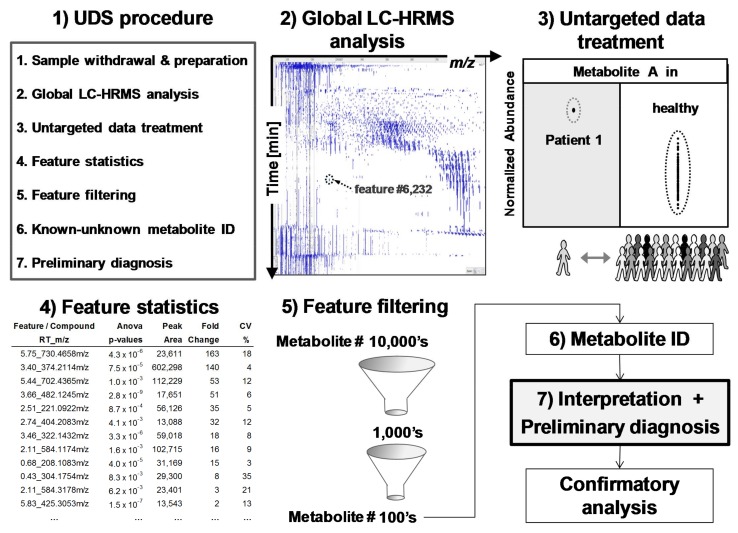
Untargeted diagnostic screening (UDS): (**1**) Overview of UDS prototole (**2**) serum extracts are analyzed by global LC-HRMS acquisition; (**3**) data treatment reveals and ranks, via (**4**) feature statistics, compounds of interest in a sick patient (test sample) in comparison to healthy population (control samples); features are (**5**) filtered and (**6**) identified for (**7**) biomedical interpretation and preliminary diagnosis prior confirmatory, targeted, and quantitative analyses. A feature is an ion-retention time entity that corresponds roughly to a compound.

The feasibility of the UDS procedure (sample preparation, untargeted data acquisition, and treatment) is described here. This procedure needs more than 6 h and up to a few days if confirmatory analyses have to be performed. Thus, UDS is not applicable for emergency purposes. Based on high-resolution mass spectrometry (HRMS) and full-scan analysis, UDS takes into account all detected metabolites, is mainly data-driven, and, therefore, is a metabolomics analysis that needs strong software support for data treatment (Figure 3). Metabolomics, defined as the determination of the metabolome, is particularly interesting because it can reveal unexpected (1) xenobiotics in chronic or acute intoxications (toxicology screening) or (2) endogenous metabolite levels that reflect the true biological status of many human diseases including genetic variants [18,19,20,21,22,23]. Metabolites are (1) xenobiotics and (2) small endogenous molecules biosynthesized by the organism or its microbiota [23].

Metabolomics is a technology-driven field [24] and is able (1) to determine 10,000’s of features (*m*/*z*-retention time pairs) in the same analysis by untargeted acquisition and (2) to compare detected features/metabolites from sick (test) and healthy (control) people using data treatment performed by dedicated computer programs [10,18,19,20,24,25]. Figure 3 depicts a typical metabolomics workflow (see online Supplementary Materials Figure S1 for more detailed steps).

For a few decades, the analysis of the metabolome has been performed in clinical chemistry labs but with targeted analyses of <50 compounds (Figure 1). However, the recent improvement of analytical tools and especially of HRMS [24,26,27] allows for the performance of more comprehensive determinations of the metabolome [28]. Today’s HRMS instruments show similar quantitative performance when recording full-scan acquisitions (sensitivity, accuracy, precision, robustness, ease of use, etc.) than triple quadrupole instruments when recording ion transition acquisitions, allowing for a paradigm shift in LC-MS analyses and in various fields [26,29,30,31,32,33]. Thus, with sensitive and selective liquid chromatography (LC)-HRMS analysis, a much larger coverage of metabolites can be considered. In this context, UDS should be seen as the logical extension of targeted monitoring/analyses [11,34,35,36,37]. In addition, UDS approaches have already been conceived but were rather a possible/probable extension of targeted analyses or perspectives [5,7,38,39,40]. However, a key question remains when considering only the untargeted approach and the comparison between one patient’s metabolome with healthy metabolomes: could UDS be performed as a routine analysis?

In this article, we have investigated the use of metabolomics for untargeted diagnostic screening (UDS). Different test serum samples were spiked with only one xenobiotic or a higher level of one endogenous compound and compared with control samples (Figure 2). The goal was to reveal the spiked compound in each test sample metabolome (considered as the patient’s metabolome) using an untargeted approach in order to study the feasibility of the UDS procedure for routine analysis. The further goal would be to establish a preliminary diagnosis in relation to the patient’s symptoms and the uncovered metabolites, but this cannot be done with the samples of this feasibility study. However, with known molecules spiked in the plasma samples, we know the results which should be found. Bottlenecks, biases, and key criteria for the realistic application of UDS in clinical labs can be evaluated in depth.

UDS could be performed to uncover new diagnosis hypotheses when canonical processes do not reveal disease etiology nor establish a clear diagnosis. On the other hand, the risk of overdiagnosis associated with possible false positive hits is discussed.

## 2. Materials and Methods

### 2.1. Biomatrix and Sample Preparation

Human serum samples were prepared from anonymized patients’ whole blood withdrawn in collection tubes (Sarstedt^®^ Monovettes, Numbrecht, Germany). Two hundred microliters of serum were extracted by protein precipitation using 3 volumes of methanol and centrifuged at 20,000 *g* for 12 min at 4 °C. Supernatants were transferred, dried under N_2_ flux, reconstituted with 100 μL of H_2_O:acetonitrile solution (*v*:*v*, 3:1), and placed in polypropylene injection vials. No internal standards were added in the samples prior to extraction. For the test samples, two reconstituted extracts were pooled because of volume constraints and spiked with a pure standard solution containing: (a) methadone, (b) methamphetamine, (c) dextromethorphan, (d) endoxifen (a metabolite of tamoxifen), (e) imatinib, (f) DHEA-S (dehydroepiandrosterone-sulfate), or (g) testosterone with the following final concentrations: (a), (b), and (c) 5 ng/mL; (d) 0.5 and 5 μg/mL; (e) 5 μg/mL; (f) 1, 5, and 20 μM; or (g) 17.5, 35, and 70 nM (Table 1). Endogenous and exogenous compounds were chosen based on their availability in our lab. Environmental chemicals or food additives were not tested, but the UDS procedure can be applied to all compounds as far as they are extracted and ionized by the ion source. Concentrations were chosen to be low, medium, or high corresponding to higher levels of the endogenous levels and to chronic or acute intoxications. Test samples contained one spiked compound only. In contrast to xenobiotics (a–d), serum extracts contained endogenous levels of DHEA-S (e) and testosterone (f) and, therefore, final concentrations have to take into account both endogenous (unknown) and spiked amounts (e.g., DHEA-S = [endogen.] + 5 μM).

### 2.2. LC-HRMS System, Parameters, and Analysis

The chromatographic system consisted of an LC pump (Rheos-Allegro, Flux, Basel, Switzerland), an autosampler (CTC Analytics AG, Zwingen, Switzerland) maintaining injection vials at 10 °C, and a Q-Exactive Focus^®^ HRMS (Thermo, Waltham, MA, USA) controlled by Xcalibur^®^ (Thermo, Waltham, MA, USA). Extracts were injected onto BEH C18 guard and analytical columns (Waters, Milford, MA, USA), 10 and 30 mm × 2.1 mm (L × i.d.) with 1.7 µm part. size. The mobile phase was composed of MS grade (A) water and (B) acetonitrile, both with 0.1% FA and delivered at 0.6 mL/min. The ion source was a heated electrospray source (H-ESI type II) performing in positive or negative mode with sheath and auxiliary gas flow set at 35 and 25 arb. units, spray voltage set at 3200 V, and auxiliary gas and capillary temperatures set at 320 °C and 400 °C, respectively. The stepwise gradient was delivered as follows: 0–0.5 min, 95% of phase A; 0.5–10 min, 95–5% of phase A; 10–12 min, 5–2% of phase A; and 12–15 min, 95% of phase A for a 15 min total run time. The injection volume was 10 μL. HRMS full scans were acquired from *m*/*z* 60–900 Da in profile mode for the first 13 min (mean acquisition rate, measured on the complete run, was 2.7 scans/s). The automatic gain control and mass resolution were set at 1 × 10^6^ ions and 70,000 (*m*/*z* = 200), respectively. LC-HRMS analyses were performed with external mass calibration that was performed once a week and prior to this analysis.

The metabolome of one test sample (containing the spiked molecule and considered as the patient’s sample) was compared with healthy metabolomes of the control group. Test samples were injected in triplicate (*N* = 3) and compared with two types of control samples: (1) a pool sample (Pool) prepared from 25 different individuals and injected in triplicate (*N* = 3) or (2) 95 unpooled samples (N95) prepared from 95 different individuals and injected once each (*N* = 95; 95 single injections) (see Figure 2). Control samples were injected between each test sample. Memory effect was verified with blank samples and remained below ~2%. Sixty hours of analysis were performed in negative and positive mode as follows: 48 h corresponding to the N95 control samples, followed by ~12 h of, alternatively, the control Pool and test samples.

### 2.3. Data Representation and Data Treatment

HRMS full-scan data were treated with Progenesis^®^ QI metabolomics software (version in 2014, Nonlinear Dynamics, Newcastle, UK) for untargeted data treatment and with Xcalibur^®^ (Thermo, USA) for targeted determinations. Extracted-ion chromatograms, XIC, were constructed using Xcalibur^®^ and used a mass-extraction window set at ±10 ppm, centered on the theoretical *m*/*z* (*m*/*z_theor_*) or, for unknown ions, on measured *m*/*z* (*m*/*z_meas_*). Metabolite LC-HRMS peak areas found with Progenesis^®^ or Xcalibur^®^ were compared. Raw files were imported from Xcalibur^®^ to Progenesis^®^ software with a filter strength set at 1.0 (default value). Then, features were automatically (1) aligned, (2). picked with “default” Progenesis^®^ detection settings and peak widths set at ≥0.04 min, (3) Normalized, and (4) grouped (deconvolution) based on the following adducts: +[H_2_O+H^+^]^+^, +[H^+^]^+^, +[2H^+^]^2+^, +[NH_4_^+^]^+^, +[Na^+^]^+^, and +[K^+^]^+^ or −[H^+^]^−^, −[H_2_O-H^+^]^−^, −[2H^+^]^2−^ for positive or negative mode. Positive or negative data were treated separately. Raw peak areas were normalized by the sum of all feature peak areas following one classical Progenesis^®^ procedure. For each sample, peak alignment and area normalization were verified by looking at their associated Progenesis^®^ values. Progenesis^®^ software compared a test sample (triplicate injections) with Pool or N95 control samples (triplicate or simplicate injections, respectively). Thus, the Progenesis^®^ study design compared 3 versus 3 or 3 versus 95 samples (see Figure 2). Please refer to the Supplementary Materials Figure S1 for a more complete data processing representation.

### 2.4. Evaluation of Untargeted Diagnostics Screening

From more than 10,000’s detected and listed features, six different filters were applied to remove irrelevant features and reveal the spiked compound more easily. These filters considered different parameters/statistics provided by Progenesis^®^ (filters #1 to #4) or calculated in Excel^®^ spreadsheet (Microsoft, San Francisco, CA, USA; filters #5 and #6) after importation of the remaining features filtered by filter #1 to #4.

Features were removed (filtered) when: (#1) the mean peak areas in the test sample (*N* = 3) were less than in the control samples (Pool or N95); (#2) CV values of the feature peak area in the test sample were >25% (variation of three injections); (#3) *p*-values (ANOVA test) between test and control groups were >0.005; and (#4) fold differences between the mean peak area of test and control groups were <5. After the application of these four filters, the remaining number of features was <400. This smaller number of features was suitable to be exported from Progenesis^®^ into Excel^®^ for further filter application.

**Figure 4:**
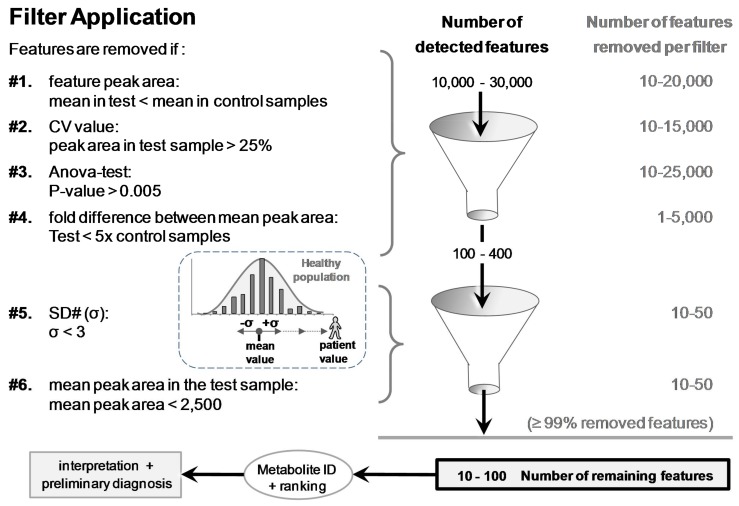
The six filters used to discard ≥99% of the >10,000 detected features. Remaining features were eventually ranked based on SD# and mean peak area values prior to putative identifications and biomedical interpretations. SD# value was calculated with the following equation: SD# of feature *f* = [(|peak area*^f^*_mean_ in test sample − peak area*^f^*_mean_ in control group|)/(SD of control group)]. It corresponds to the distance, as a number of [σ] (=SD#), between the peak area mean of the test and the peak area mean of the control samples (e.g., samples of a patient and a healthy population).

The last two filters, #5 and #6 applied in Excel^®^, reduced further the number of features while enabling a final ranking. Filter #5 removed features showing SD# (σ) < 3.0; SD# corresponds to the delta of the feature mean peak area in the test and control samples, divided by the standard deviation in the control group (e.g., SD# of feature *f* = [(|peak area*^f^*_mean_ in test sample − peak area*^f^*_mean_ in control group|)/(SD of control group)] (see Figure 4). Filter #6 removed features with peak areas that were <2500 Progenesis^®^ arbitrary units in test samples (equivalent to 100,000 Xcalibur^®^ arb. units; see Supplementary Materials Figure S2). The choice to remove these small peak areas was made to discard unreliable/irreproducible detections and artifacts (e.g., trace levels, noise, and electronic spikes).

Supplementary Materials Figures S3 and S4 depict feature parameters calculated by Progenesis^®^ and SD# calculation. We have postulated that normal distributions of feature peak areas were an acceptable approximation (see typical distributions of feature peak areas in Supplementary Materials Figure S5). Finally, in Excel^®^, the remaining features were ranked from highest to lowest (1) SD# or (2) peak area values prior to putative identification (see Supplementary Materials Table S1).

### 2.5. Identification of Revealed Features

The last remaining features were putatively identified by matching *m*/*z_meas_* with reference entries in the Human Metabolome Database (HMDB, www.hmdb.ca, [41]). Only specific adducts (see Section 2.3 Data Representation and Data Treatment) with a mass accuracy of ≤±5 ppm were allowed to return possible compounds. Therefore, only known-unknown metabolites from a “small size” database, HMDB, were considered (<0.5 × 10^6^ entries like Metlin or KEGG databases [42,43]). Big databases (>50 × 10^6^ entries), such as ChemSpider, PubChem, CAS registry, etc. [44,45,46], were not considered here because most compounds registered in these big databases have an extremely low probability to be found in human plasma [47]. Identification confidence of the putatively identified known-unknown metabolites was evaluated based on a recent publication that proposes the use of an identification (ID) confidence scale and ID score [47]. These ID scores are based on a relative retention time or not, and the collection of ID points from various criteria such as mass accuracy, relative isotopic abundance, fragment ions, fine isotopic distribution, etc. [47].

## 3. Results and Discussion

### 3.1. Data Treatment and Reliability

The reliability/robustness of sample extraction, LC-HRMS analysis, and data treatment was verified using the difference between samples of the retention time alignment values and of the peak area normalization values (based on the sum of all feature peak area). Out of ~250 samples injected and based on these alignment and normalization values, no drop-out samples/analyses were observed and removed. In addition, simple comparisons of peak area, mass accuracy, relative isotopic abundance, and retention time values, determined with Progenesis^®^ or Xcalibur^®^ computer programs, showed similar results. A typical correlation between peak areas obtained with both softwares (untargeted and targeted, respectively) is shown in Supplementary Materials Figure S2. Whereas the peak area values differ by 37 between the targeted and untargeted softwares as the result of different arbitrary units, excellent correlations (*R*^2^ > 0.9) were observed. 2D-gel and XIC representations with, respectively, Progenesis^®^ and Xcalibur^®^, are depicted in the online Supplementary Materials Figure S6. These comparisons sustain the reliability of untargeted data treatment with metabolomics softwares.

### 3.2. Applied Filters for Feature Removal and Revealed Metabolites

Feature-associated parameters (peak area, *p*-values, etc.) allowed for applying filters to discard ≥99% of the 10–30,000 detected features (Figure 4). Removed features represented 66%, 50%, 83%, and 17% when the following filters were individually applied, respectively: (#1) mean peak area value in control samples > in the test sample, (#2) CV of the mean peak area in the test sample >25% (*N* = 3 injections), (#3) *p*-value between groups >0.005, or (#4) fold difference of the mean peak area between test and control groups <5. Each of the last two filters, (#5) SD# <3 and (#6) peak area <2500 arb. units, removed an additional ~10–25% (Figure 4). The last two filters allowed for ranking the remaining features for easier metabolite discrimination (see the section thereafter).

**Figure 5:**
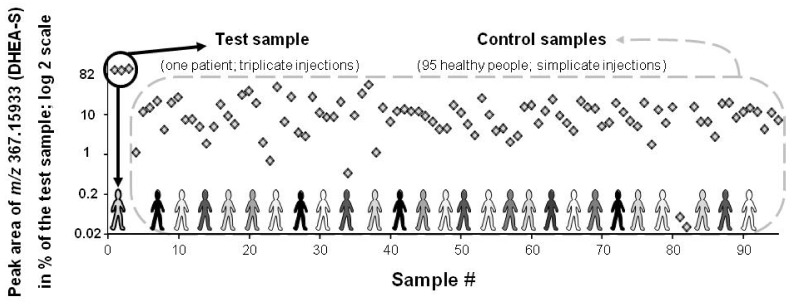
Peak area of a spiked feature (DHEA-S: [endogen.] + 20 μM; *m*/*z* measured at 367.15933) in the test and control serum samples (black circle and dashed grey line box, respectively). Results are expressed as [% of the test sample] with a log 2 scale. In serum, DHEA-S population value ranges from 0.4 to 12 μM (population diversity exemplified with various shades of grey). DHEA-S is ranked at the 1st and 25th position when, respectively, peak area or SD# are taken into account.

Filter #1 was used to find chronic or acute exposure to xenobiotics or higher levels of endogenous metabolites. Filter #2 was appropriate because CV values of each spiked compound were <25%, whereas different peak intensities were observed (Table 2 and Table 3). The only exception is methamphetamine (CV = 97%; Table 3), which was detected as a trace intensity in the void volume (mean peak area = 1190 arb. units; *N* = 3; [1260 + 2305 + not detected]; also removed by filter #6). Filters #3 and #4 (*p*-value and fold difference) were appropriate because none of the spiked compounds were discarded. Of note is that, when N95 control group was used, filter #5 (SD#) appeared more appropriate than filter #4 (fold change) because SD# takes into account the dispersion of the feature/compound peak area (see Table 2 and next section). Figure 5 (for more details, please see Supplementary Materials Figure S7) and Supplementary Materials Figure S8 show, respectively, the peak area of two spiked metabolites, DHEA-S, and endoxifen, and of the ~50 features that remained after filter application. Both spiked metabolites (in two different test samples) were well uncovered in the metabolome of the test sample(s) when compared with the N95 control samples (95 healthy metabolomes). However, with testosterone, which has a gender-bimodal distribution, the use of a normal distribution with the entire population resulted in false negative outcome in contrast to the use of a female or male subpopulation (showing lower population SD and higher SD# values; see Supplementary Materials Figure S9). Thus, it appears more appropriate to use a female or male subpopulation than the entire population as a control group to reveal drop-out sex-related endogenous metabolite concentrations.

Each spiked compound was revealed and ranked in a final Excel sheet (Table 3). The exceptions were methamphetamine that was discarded for its trace level intensity and a too high CV value as well as the two lowest DHEA-S and testosterone spiked levels that were in the population range (true negative).

### 3.3. Spiked Compound Revealed with Pool or N95 as Control Groups

Table 2 shows how spiked endoxifen (0.5 or 5 μg/mL) or DHEA-S ([endogen.] + 20 μM) were revealed in each test sample when compared with the control groups (Pool = one pool sample or N95 = 95 metabolomes to determine). The spiked compound was revealed using both control groups and showed close *p*-values, fold differences in mean peak area, and SD# (Table 2). However, a clear difference was observed with the number of remaining features (30–50% less with N95 than Pool as control groups) or with the ranking based on SD# (Table 2). This confirms that a “large” control group is capable of removing a larger number of irrelevant features than one control sample of pooled individuals. However, using peak area ranking (Table 2), the Pool control sample allowed endoxifen (5 μg/mL) and DHEA-S (20 μM) to be ranked at the top. This suggests that a pool sample can be sufficient to reveal compounds of interest when “acute” intoxication is suspected. An advantage is the limited number of injections: triplicate injections for both test and Pool control samples (~2 h of LC-HRMS analysis in positive mode) in contrast to a large population (95 control samples; ~30 h of LC-HRMS analysis in positive mode); see Figure 2. Nevertheless, the use of N95 as a control group appears more adequate for both endogenous metabolites and xenobiotic screening. It would be cost-effective if, every week, ≥~50 patients’ samples are screened and if they are injected between the control samples. UDS results are given as relative values of the control samples (healthy people metabolomes). Therefore, the control group should be described as best as possible (age, sex, etc.) in order to get the best translatable and universal results.

### 3.4. Feature Ranking Based on SD# or Peak Area

Table 3 shows, after filter application based on N95 as the control group, the ranking of the spiked compound in the final list of remaining features. The two different rankings, based on (1) SD# or (2) feature peak area in the test sample, have both advantages and disadvantages. When the ranking is based on the peak area, high levels of xenobiotics corresponding to “acute” intoxications or recent food-product intake would logically be found on the top of the final list. This can be observed in Table 3 with endoxifen and imatinib, both spiked at 5 μg/mL. When SD# is used to rank the features, there is a risk to overestimate trace peak areas that are present in the test sample but mainly absent in the control group. In our analyses, these revealed compounds were false positive hits and are likely related to recent diet consumption or natural environmental exposure. However, compounds showing low signal intensities (low ionization yield or low concentrations) can be better revealed (top ranking) using the SD# ranking as shown in Table 3 with methamphetamine and methadone, both spiked at 5 ng/mL in different samples.

It appears pertinent to evaluate both peak area and SD# ranking and, importantly, to identify the remaining features in order to relate the identified compound(s) to the patient’s symptoms. Indeed, identification of the remaining features is crucial because the results obtained with this UDS procedure must be interpreted in light of biomedical knowledge and the patient’s problems. This should be considered as the final and key filter of untargeted diagnostic screening prior to confirmatory, targeted, and quantitative determination(s) of the incriminated compound(s)/enzyme(s). A proper biomedical interpretation and pertinent association between compound(s) revealed by UDS and the patient’s symptoms are the barriers against overdiagnosis.

### 3.5. Metabolite Identification

Metabolite identification is widely recognized as a bottleneck in metabolomics [48]. This is particularly true when metabolites are unknown-unknown compounds (metabolites not present in usual databases) or in source fragments. However, a strong effort has been made to improve rapid and automatic metabolite identification of known-unknown metabolites (metabolites with their ID number in usual databases). The identification can rely on various LC-HRMS parameters such as relative retention time, mass accuracy of the precursor or fragment ions, relative isotopic abundance, fine isotopic distribution, etc. Today, metabolite ID relies on two main references [49,50]. The first reference demands the availability and injection of pure standards, whereas the second gives very general ID categories. Recently, an ID scale and ID score have been proposed to help in the confidence of metabolite identification [47]. Metabolite identification is usually the most time-consuming step. However, the newest versions of metabolomics software allow more and more automatic metabolite ID and will make the UDS procedure more and more realistic and cost-effective. Here, putative identifications were carried out manually using *m*/*z_meas_* accurate mass as a search parameter in HMDB [41] (see mass accuracy tolerance and chosen adducts in Section 2 Materials and Methods).

Supplementary Materials Table S1A depicts an example of putative identifications of unknowns in the final list of features. According to a recent identification confidence scale and score [47], feature identifications were fair, annotated, or unidentified (ID score: 2D3, 3D, and 4, respectively). In this example, where the sample was spiked with endoxifen and compared to the N95 control group, endoxifen was revealed and putatively identified with an ID Score of 2D3 (fair identification [47]), providing enough confidence to consider further confirmatory, quantitative, and targeted quantifications if needed. We believe that putative identifications can be reliable enough to relate uncovered metabolites and symptoms and to ask for a confirmatory analysis.

As exemplified with the Supplementary Materials Table S1A, more than 50% of the remaining features were unidentified, most (two-thirds) were annotated, and a few (one-third) were putatively identified using mass accuracy, relative isotopic abundance (e.g., triamcinolone, a corticosteroid drug; trihydroxy-methyl-diprenylxanthone, a fruit constituent; drotaverine, an antispasmodic drug; and endoxifen, the spiked metabolite of tamoxifen; see Supplementary Materials Table S1B). Annotated features suggest that, in the test samples, many revealed metabolites are coming from food intake and other usual environmental exposure. In the UDS procedure, the identification work would logically be the work of the bioanalysts, who will establish a list of metabolite outliers, putatively identified. Then, relation(s) between clinical symptoms and metabolite outlier(s) should be done by clinicians (MDs or biochemists). If the relation(s) would appear to be relevant for new diagnosis assumptions, confirmatory analyses should be performed (e.g., targeted and absolute quantifications of the metabolites).

## 4. Conclusive Remarks

Today’s HRMS instruments, recording full scans, show similar quantitative and semiquantitative performance to triple quadrupole recording ion transitions [26,29,30,31,32,33]. This allows a paradigm shift where the exact same instrument can routinely perform targeted or untargeted investigations. One could argue that the evaluated UDS procedure is not appropriate for very hydrophilic or lipophilic compounds. However, the tested procedure could be used with an HILIC column, an extraction that is specific to lipids, or other ion sources that are more capable of ionizing some pollutants [51,52,53]. This would be the “only” additional UDS procedures. The proposed UDS, using protein precipitation with methanol and LC-HRMS analysis with a C18 column, is probably the most global procedure today [10].

Importantly, the UDS procedure has to be based on robust sample withdrawal, preparation, and storage prior to extraction because strong biases can be associated to these first steps. Here, sample withdrawal, preparation, and storage followed strict but usual procedures in hospitals. No drop-out samples were observed underlying the reproducibility of the preanalytical procedure as well as the LC-HRMS analysis.

Using global untargeted acquisition and unsupervised data treatment, the spiked xenobiotic and higher endogenous metabolite levels were revealed in their serum extract after the removal of ≥99% of detected features and their ranking based on peak area or SD#. Metabolites revealed by the UDS procedure in the test (“patient’s”) sample together with the relation to patient’s symptoms could help to establish a diagnosis hypothesis when canonical procedure fails. The most appropriate control group is a subpopulation of healthy male or female individuals because UDS results are given as relative values of the control group. Therefore, a well-defined control group (age, sex, etc.) will allow universal understanding of the patient’s UDS results.

LC-HRMS analysis and data treatment appear realistic and cost-effective and support the claim that UDS is feasible and appropriate when a canonical diagnostic process does not allow for establishing a clear diagnosis (Figure 1). In the near future (<10 years), we could foresee a UDS procedure with ≥50 patients and ≥100 control samples per week with both positive and negative detections. UDS could help in discovering a new diagnosis hypothesis and in reducing misdiagnosis by revealing unexpected xenobiotics or higher endogenous levels. The risk of overdiagnosis of this approach should be reduced by mandatory biomedical interpretation of UDS preliminary results with the patient’s symptoms (the final filter to be applied) and by confirmatory, targeted, and quantitative determinations (e.g., diseases severity, subphenotypes, and/or compound identity verification). With the anticipated implementation of fully automated MS analyzers, such as the Cascadion^®^ system, in various labs such as clinical labs, there will be some free space for more demanding and personalized determinations, such as the UDS procedure.

While we believe that our feasibility study has built a foundation for UDS procedure(s), further evaluations and improvements are needed. These would be: to test UDS with real undiagnosed patients’ samples; to better evaluate standardization and signal normalization; to acquire MS/MS spectra in parallel to HR full scan to improve feature identification (e.g., product ions from data independent or data dependent acquisition); to perform UDS on a longer period of time (e.g., months rather than a few days); to establish a fully automated metabolite identification and ranking; to evaluate the success rate in uncovering new diagnosis hypotheses; and to estimate the number of medical requests for this screening and metabolomic analysis (medical pertinence).

Finally, considering that patients and their metabolomes should be considered as unique entities and not necessarily belonging to a group that is predefined by guidelines [16,17], the huge biological variability of diseases is better addressed with the UDS procedure because a patient’s metabolome is compared to 95 healthy metabolomes representing the control population (Figure 2 and Figure 3).

## Figures and Tables

**Table 1:** Chemical information on the spiked compounds (*).

**Xenobiotic**	**Formula**	**Adducts**	***m*/*z_theor_* [Da]**	**[μg/mL]**	**LC Area ^◊^**	**RT [min]**	**MA [ppm]**	**CV [%]**
methamphetamine	C_10_H_15_N	+H^+^	150.12773	0.005	1	0.4	0.7	97
methadone	C_21_H_27_NO	+H^+^	310.21654	0.005	64	3.2	−0.2	22
dextromethorphan	C_18_H_25_NO	+H^+^	272.20089	0.005	76	2.6	−0.2	5
endoxifen	C_25_H_27_NO_2_	+H^+^	374.21146	5/0.5	6778/602	3.4	−0.1/0.5	6/4
imatinib	C_29_H_31_N_7_O	+2H^+^	247.63678	5.0	159,359	1.9	0.3	10
**Endogenous**	**Formula**	**Adducts**	***m*/*z_theor_* [Da]**	**[μM]**	**LC Area ^◊^**	**RT [min]**	**MA [ppm]**	**CV [%]**
DHEA-S	C_19_H_28_O_5_S	−[H^+^]^−^	367.15847	20 ^○^	61,361	3.2	2.4	5
testosterone	C_19_H_28_O_2_	+H^+^	289.21621	0.07 ^○^	91	3.4	−0.4	6

(*) only one compound was spiked per test sample. (^◊^) LC-HRMS peak area determined with Progenesis^®^ and given as normalized arb. units × 10^−3^. (^○^) added level to the endogenous level—see Section 2 Materials and Methods. Two lower amounts were also spiked but not revealed in the UDS procedure because their levels were not different to those of the control. Abbreviations: DHEA-S, dehydroepiandrosterone sulfate; RT, retention time; MA, mass accuracy; CV, coefficient of variation in the test sample (triplicate injections).

**Table 2:** Spiked compounds in the test sample compared to two different control groups: a pooled sample (Pool; 1 sample injected in triplicate) or 95 different healthy individual samples (N95; injected in simplicate).

**Control**	**Spiked: Endoxifen (Xenobiotic)**			Fold Difference **			**Ranking # Based on**	
**Group**	**[μg/mL]**	LC Area *	**CV [%]**	**Anova (*p*)**	LC Area *	**SD#**	**SD#**	LC Area *
N95	0.5	602	3.7	0.0001	140	101	3	11
Pool	0.5	509	4.9	0.0048	186	118	127	12
N95	5	6778	5.5	<0.0001	1205	900	13	1
Pool	5	6365	4.6	0.0012	1927	1259	55	1
**Control**	**Spiked: DHEAS (Endogenous)**			Fold Difference **			**Ranking # Based on**	
**Group**	**[μM]**	LC Area *	**CV [%]**	**Anova (*p*)**	LC Area *	**SD#**	**SD#**	LC Area *
N95	20	61,361	5.0	0.0002	9.2	10.7	25	1
Pool	20	56,819	5.6	0.0082	6.2	6.7	64	1

(*) LC-HRMS peak area, given as normalized arb. units × 10^−3^, is the mean value in the test sample; three injections. LC-HRMS peak areas are determined with Progenesis^®^. (**) fold difference between test and control groups is calculated with LC-HRMS mean peak area. Abbreviations: DHEA-S, dehydroepiandrosterone sulfate.

**Table 3:** Spiked compounds revealed in the metabolome of the test sample in comparison to 95 healthy metabolome samples using the UDS procedure. Fold difference between the test and control samples are given as ratio of mean LC-HRMS peak area or SD# (the number of SD [or σ] between LC-HRMS peak area). These two values are also used to rank the metabolites.

Xenobiotic	Spiked Levels		Fold Difference Related to			Ranking # Based on	
	[μg/mL]	CV [%] *	*p*-Value	LC Area **	SD#	SD#	LC Area (10^−3^)
methamphetamine	0.005	97 ^◊^	0.0001	105	45	33	191
methadone	0.005	22	<0.0001	14,162	3615	6	29
dextrometorphan	0.005	5	<0.0001	50	12,480	1	6
endoxifen	5.0	6	<0.0001	1205	900	13	1
imatinib	5.0	10	<0.0001	40,417	34,189	4	1
**Endogenous**	**Endo. + μM**						
DHEA-S	20	5	0.0002	9	11	25	1
testosterone	0.07	6	0.0020	5	4	44	12

(*) CV is calculated with LC-HRMS peak area in the test sample (triplicate injections). (**) fold difference between the test sample and control samples (N95) is calculated with LC-HRMS mean peak area. (^◊^) with a CV > 25% (Filter #2), methamphetamine would be the only discarded spiked compound.

## Supplementary Materials

The following are available online at http://www.mdpi.com/2218-1989/8/2/39/s1, Figure S1: Typical metabolomics workflow, Figure S2: Typical example of a correlation between LC-HRMS peak area integrated with a targeted or untargeted software, respectively, Xcalibur® and Progenesis®., Figure S3: Parameters obtained by Progenesis® for each detected feature, Figure S4: Calculation of SD#, Figure S5: Distributions of LC-HRMS feature peak area, Figure S6: Eextracted-ion-chromatogram and 2D-gel representations, Figure S7: Peak area of the DHEA-S or all remaining features in the test and control samples, Figure S8: Peak area of the endoxifen or all remaining features in the test and control samples, Figure S9: Peak area of the testosterone or all remaining features in the test and control samples, Table S1: Example of a final list of remaining compounds.

## Abbreviations

HRMS	high resolution mass spectrometry
LC	liquid chromatography
UDS	untargeted diagnostic screening
DHEA-sulfate	dehydroepiandrosterone-sulfate
ID	identification
