# Supplementary material for: LC-HRMS Metabolomics for Untargeted Diagnostic Screening in Clinical Laboratories: A Feasibility Study
Source: Metabolites. 2018 Jun 15;8(2):39. doi: 10.3390/metabo8020039 (PMC6027396; doi:10.3390/metabo8020039)
Supplement: Supplementary file 1 [file metabolites-08-00039-s001.pdf]

S1 Figure. Typical metabolomics workflow with untargeted data treatment using dedicated software use in the Untargeted Diagnostic Screening (UDS) procedure.

S2 Figure. Typical example of a correlation between LC-HRMS peak area of a feature integrated with a targeted or untargeted software, respectively, Xcalibur® and Progenesis®. Peak area are expressed in arbitrary units that are different between both softwares. Nevertheless, the curve shows an excellent coefficient of correlation.

S3 Figure. Typical parameters obtained by Progenesis® for each detected feature (retention time – *m/z* pair) in test and control samples. Raw LC-HRMS peak areas were normalized using the sum of all detected features in the sample analysis.

S4 Figure. Calculation of SD# (number of σ) based on the difference of the mean peak area of test and control samples, and the SD of the control samples (N95 = 95 control metabolomes). See equation in the dashed line box.

S5 Figure. Observed distributions of LC-HRMS normalized feature peak area. Mean value (μ) ± 3x SD (σ) corresponds to 99.7% of the entire distribution/population. SD# ≥ 3 was used as a filter to discard irrelevant features.

S6 Figure. Typical extracted-ion-chromatogram (XIC; Xcalibur® software; left) and 2D-gel representations (Progenesis® software; right) of imatinib, which was detected in a serum extract by a global LC-HRMS analysis recording in full-scan. Top and middle chromatograms reveal various adducts ($(m+Na^+)^+$, $(m+H^+)^+$, etc.) and isotopes (A, A+1, etc.) of imatinib. The bottom left table shows the relative isotopic abundance (RIA) of imatinib ($(m+2H^+)^+$) and the RIA error (measured with Xcalibur® and Progenesis® software). The bottom right XIC shows 3 in-source fragment ions of imatinib. All this data can be used for putative identification.

S7 Figure. Peak area of the spiked feature (A; DHEA-S: [endogen.] + 20 μM), or all remaining features (B; N=50) in the test and control samples (black circle and dashed grey line box, respectively). Results are expressed as [% of the test sample] with a log 2 scale.

S8 Figure. Peak area of the spiked feature (A; endoxifen: 5 μg/mL), or all remaining features (B; N=45) in the test and control samples (black circle and dashed grey line box, respectively). Results are expressed as [% of the test sample] with a log 2 scale.

S9 Figure. Peak area (Progenesis® arb. units) of the spiked feature, testosterone ([endogen.] + 17.5, 35 or 70nM) in the test and control samples (grey dots and black diamonds, respectively). Results are shown with a log 2 scale. With a male-female bimodal distribution, higher testosterone levels is better revealed with the adequate male or female subpopulation, used as controls rather than the entire population. See in the top right table, SD# and fold change values (based on mean peak area).

S1 Table. (A) Example of a final list of remaining compounds that were obtained after filter application. Here, ranking is based on SD#. (B) Three compounds were revealed in the test sample and putatively identified with usual HRMS information (accurate *m/z*, relative isotopic abundance error, etc.). Most of the revealed features are unidentified or annotated. The putatively identified metabolites could relate to patient's symptoms and deserve further confirmatory, targeted and quantitative determinations. Here, the spike compound, endoxiden is revealed and ranked at the 13th or 1st position when considering SD# or fold change between the mean peak area of test and control samples.

Figure S1

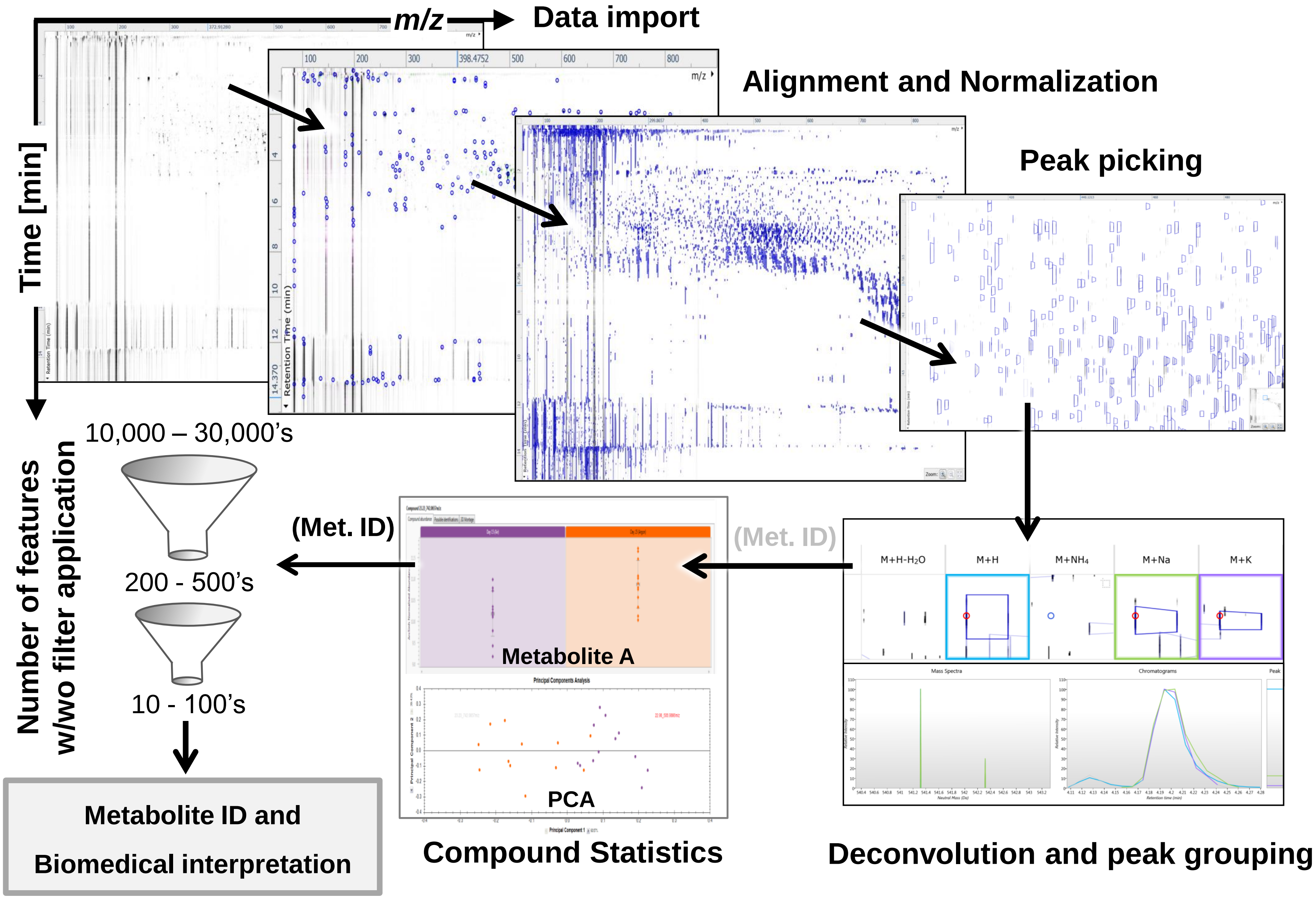
m/z
Data import
Time [min]
Alignment and Normalization
Peak picking
10,000 – 30,000's
200 - 500's
10 - 100's
Number of features w/wo filter application
(Met. ID)
(Met. ID)
M+H-H2O
M+H
M+NH4
M+Na
M+K
Mass Spectra
Chromatograms
Metabolite A
Principal Components Analysis
PCA
Metabolite ID and Biomedical interpretation
Compound Statistics
Deconvolution and peak grouping

Figure S2

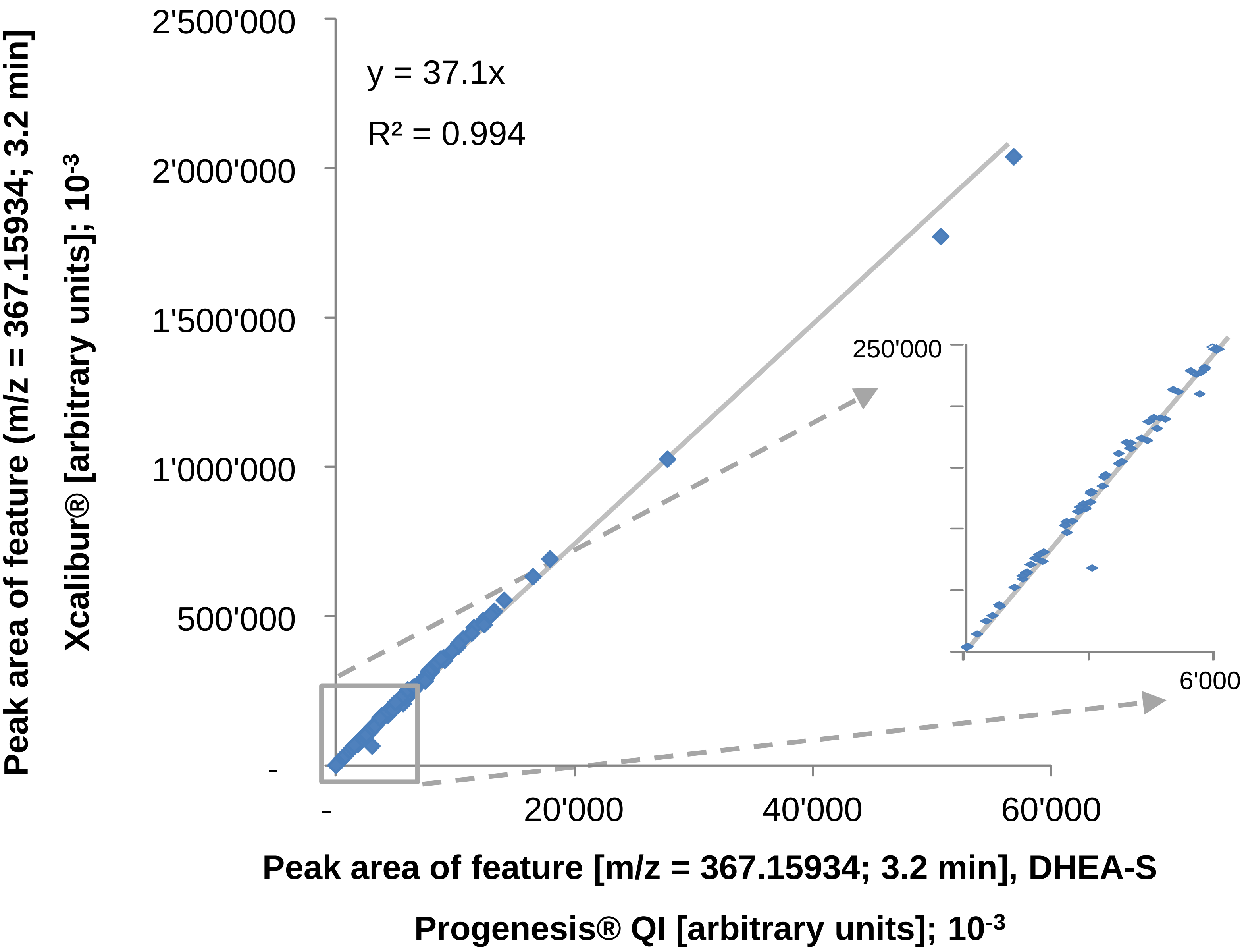
y = 37.1x
R² = 0.994
2'500'000
2'000'000
1'500'000
1'000'000
500'000
-
-
20'000
40'000
60'000
250'000
6'000
Peak area of feature (m/z = 367.15934; 3.2 min]
Xcalibur® [arbitrary units]; 10-3
Peak area of feature [m/z = 367.15934; 3.2 min], DHEA-S
Progenesis® QI [arbitrary units]; 10-3

Figure S3

Feature / Compound	m/z	z	Anova (p)	Fold Change	Isotope Distrib.	test sample CV [%]	Mean Peak Area: test	Mean Peak Area: controls	Normalised abundance: peak area (10^3) – test: inj.#1	test: inj.#2	test: inj.#3	controls: indiv.#1 →	controls: indiv.#N
RT_*m/z* values	589.305	1	4.76E-07	65.0	100 - 32.2	4.9	175	2.7	171	169	185	3.0	2.4
…	…	…	…	…	…	…	…	…	…	…	…	…	…

Figure S4

Feature / Compound	Mean Peak Area: test	Mean Peak Area: controls (●)	SD	SD# (*****)
f (RT_*m/z* values)	175	2.7	1.3	132.5
…	…	…	…	…

(●): SD is calculated from the 95 metabolomes of the control group

(*****) : SD# (or number of σ) is used to remove features (filter #5). See equation below.

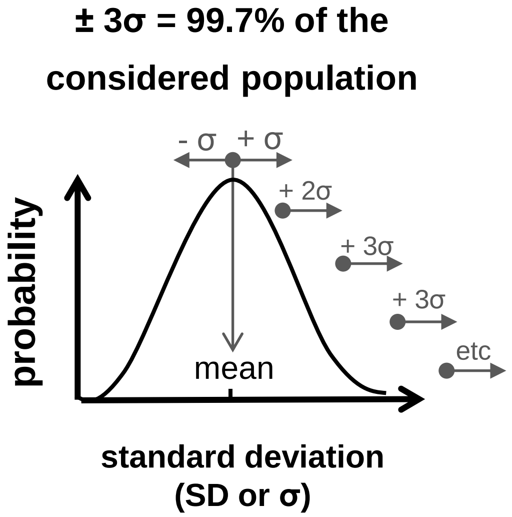

(*) SD# of feature ***f*** =

(|mean peak area^f in test sample – mean peak area^f in controls|) / SD peak area^f in controls)

Figure S5

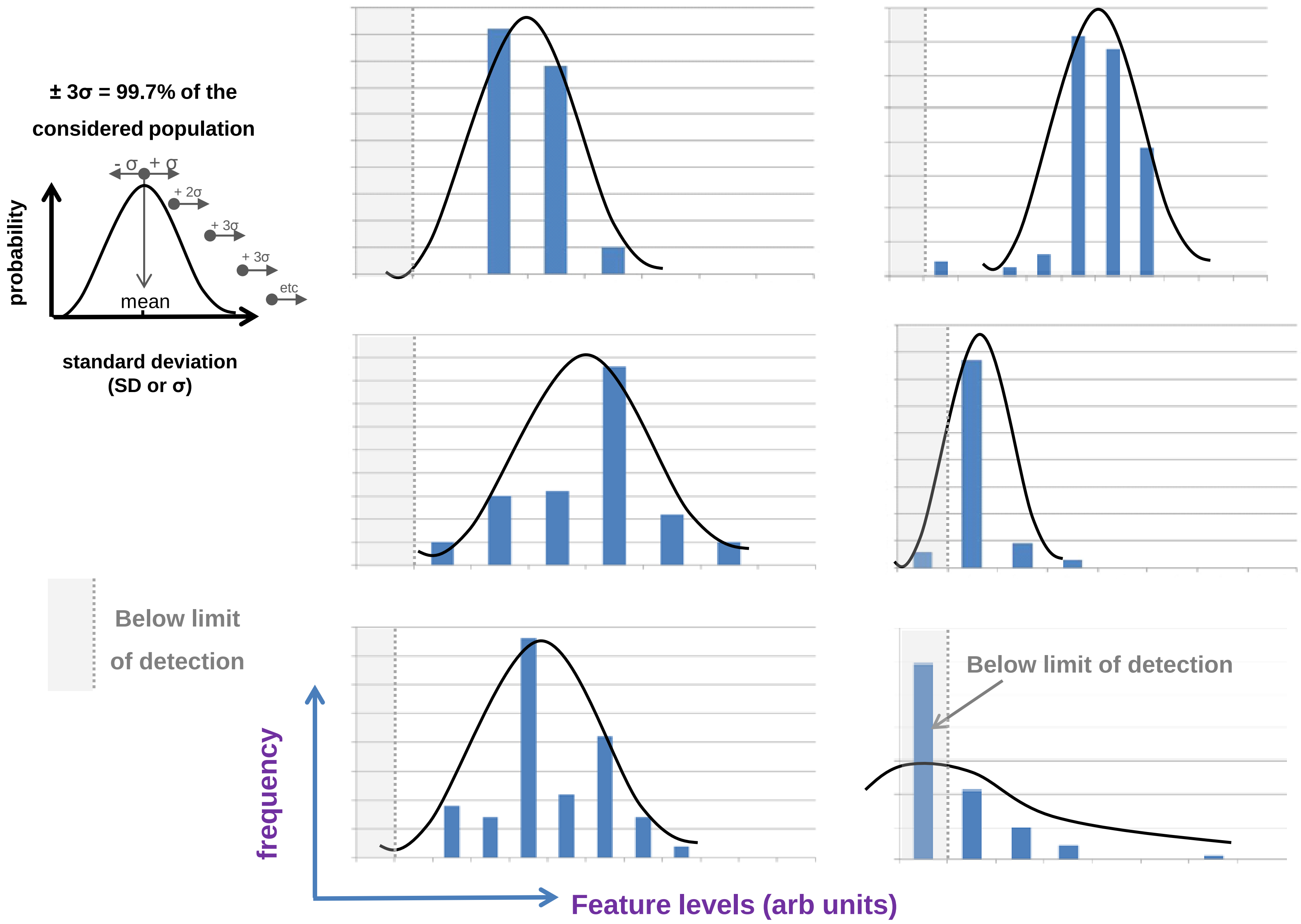
± 3σ = 99.7% of the
considered population
- σ
+ σ
+ 2σ
+ 3σ
+ 3σ
etc
probability
mean
standard deviation
(SD or σ)
Below limit
of detection
Below limit of detection
frequency
Feature levels (arb units)

Figure S6

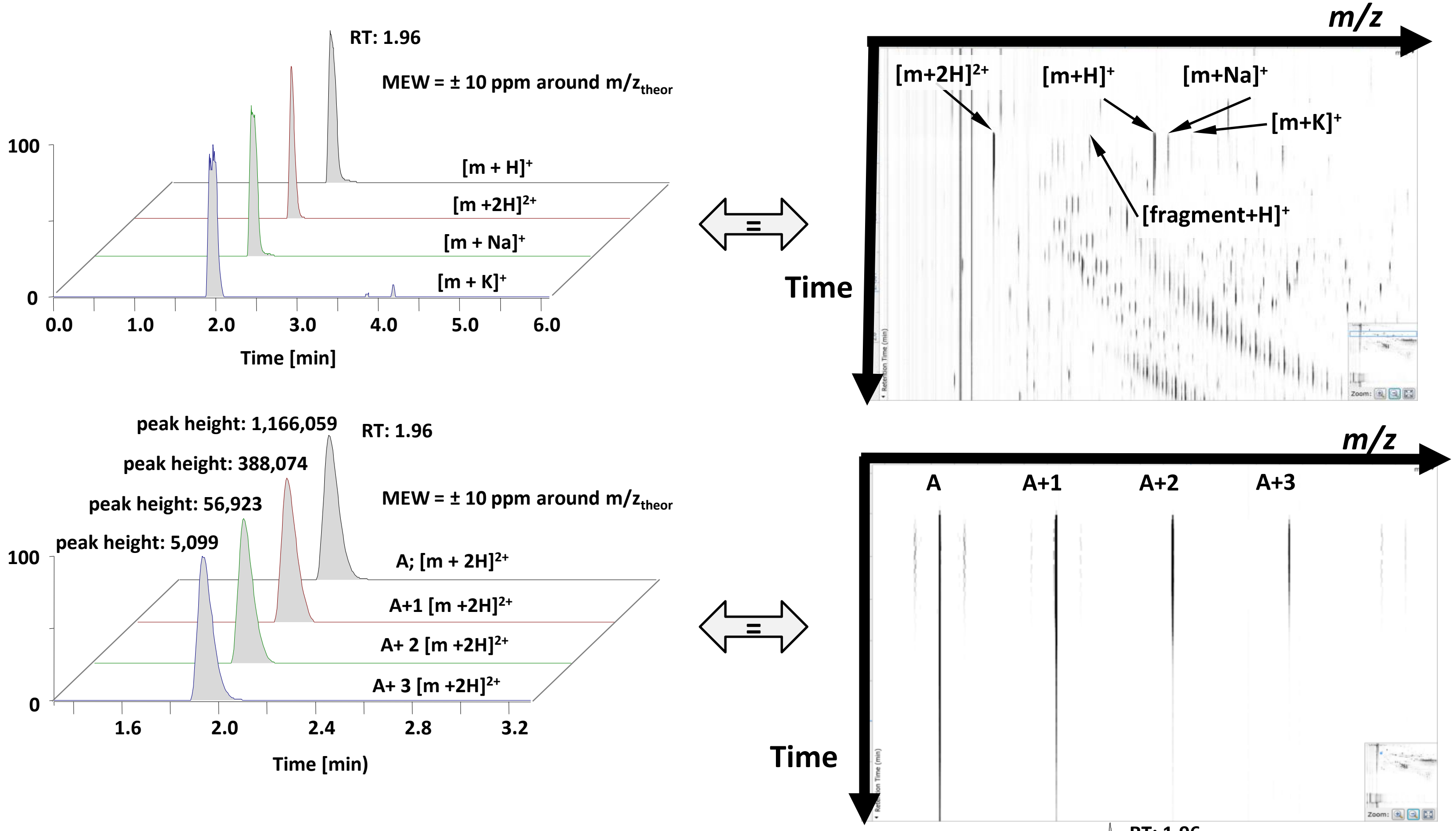

		[%] RIA error (*)	
imatinib isotope	m/z theor	Xcalibur®	Progenesis®
A; $[m+2]^{2+}$	247.6368	0.0	0.0
A+1	248.1382	-3.2	-4.9
A+2	248.6397	-17.8	-17.2
A+3	249.1411	-36.6	-53.0

(*) RIA error [%] = $(RIA_{meas} - RIA_{theor})/RIA_{theor}$) x 100

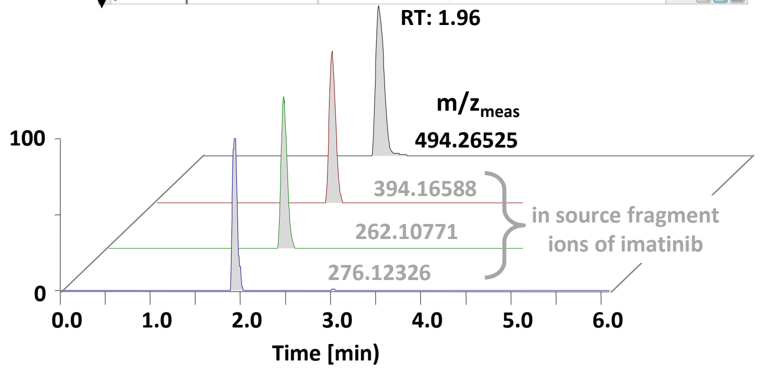

Figure S7

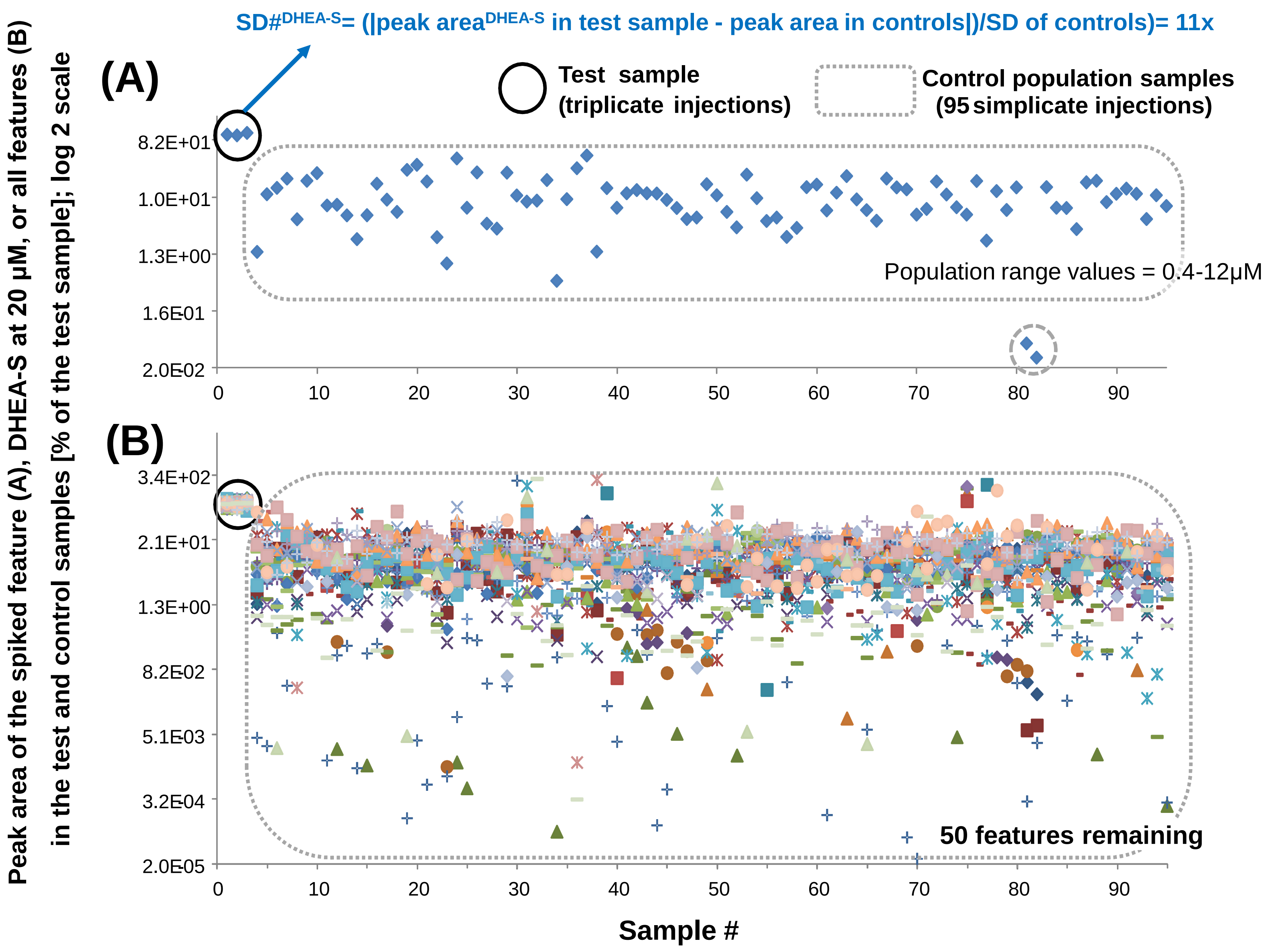
SD#DHEA-S= (|peak areaDHEA-S in test sample - peak area in controls|)/SD of controls)= 11x
(A)
Test sample
(triplicate injections)
Control population samples
(95 simplicate injections)
Population range values = 0.4-12µM
8.2E+01
1.0E+01
1.3E+00
1.6E-01
2.0E-02
0
10
20
30
40
50
60
70
80
90
(B)
3.4E+02
2.1E+01
1.3E+00
8.2E-02
5.1E-03
3.2E-04
2.0E-05
50 features remaining
Sample #
Peak area of the spiked feature (A), DHEA-S at 20 µM, or all features (B)
in the test and control samples [% of the test sample]; log 2 scale

Figure S8

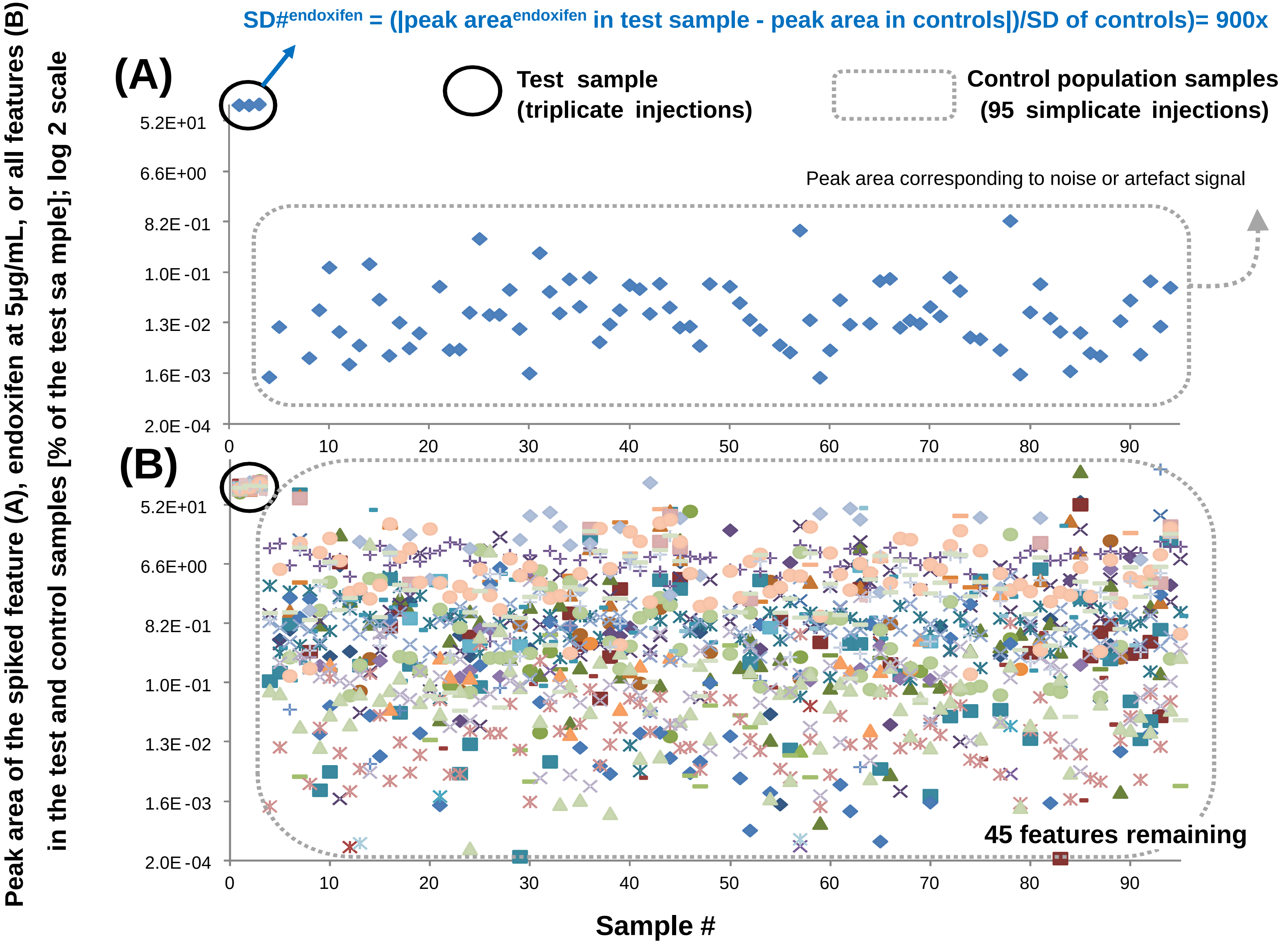

SD#endoxifen = (|peak areaendoxifen in test sample - peak area in controls|)/SD of controls)= 900x
(A)
Test sample (triplicate injections)
Control population samples (95 simplicate injections)
Peak area corresponding to noise or artefact signal
(B)
45 features remaining
Peak area of the spiked feature (A), endoxifen at 5µg/mL, or all features (B) in the test and control samples [% of the test sa mple]; log 2 scale
5.2E+01
6.6E+00
8.2E-01
1.0E-01
1.3E-02
1.6E-03
2.0E-04
0
10
20
30
40
50
60
70
80
90
Sample #

Figure S9

spiked testo. (nM)	70	34.5	17.5	70	34.5	17.5
	SD#			fold change - peak area		
entire population	3.6	2.1	0.9	4.7	3.1	1.9
female population	59.2	39.1	23.5	40.3	27.0	16.6
male population	4.0	1.8	0.1	2.5	1.7	1.0

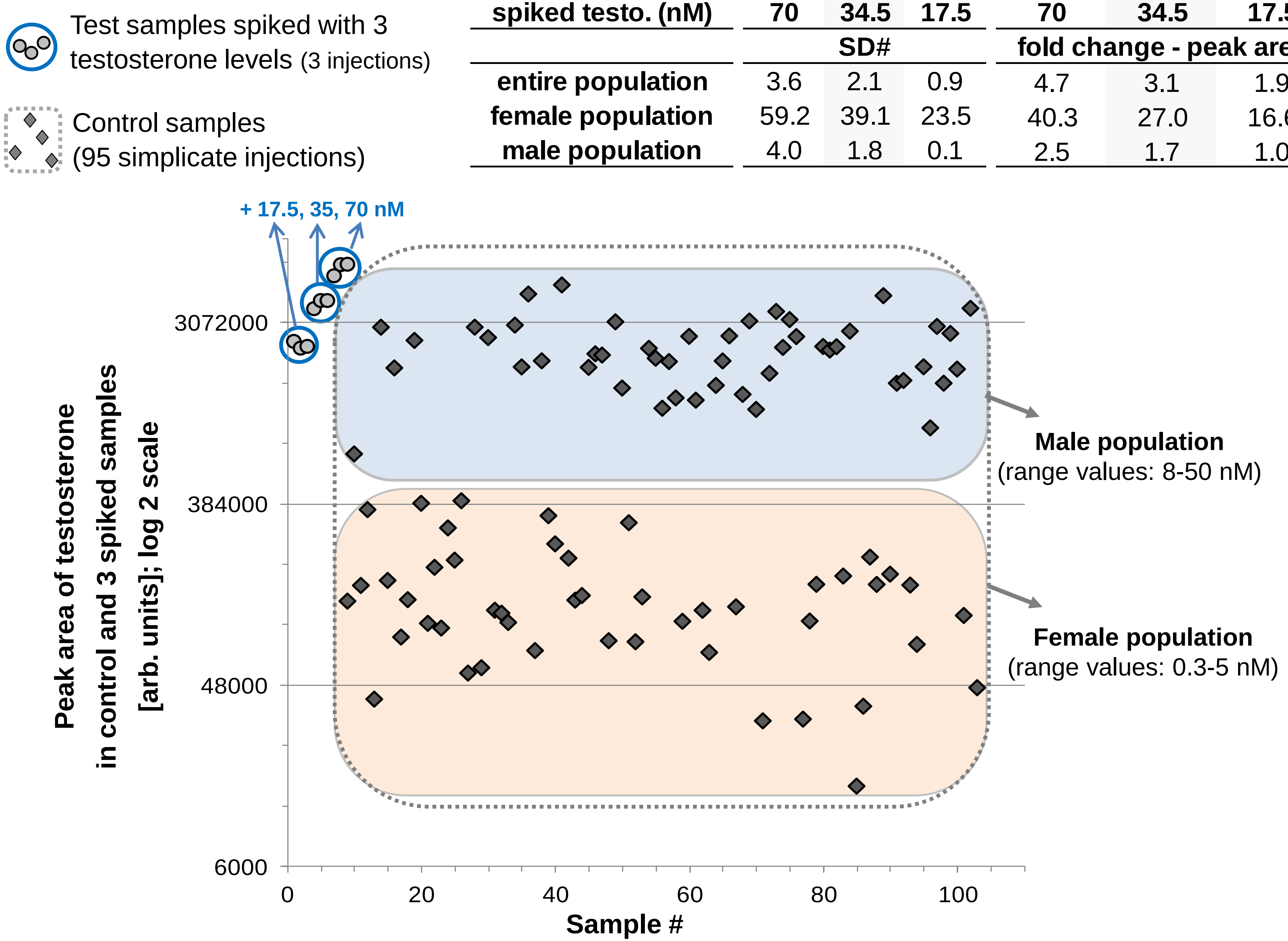

Table S1

(A)

Most probable Compound ID	compound info	m/z	z	RT	Isotope Distribution	# of SD	Spiked	N95 controls	
							Mean peak area		
Physalin	food	562.22891	1	0.6	100 - 29	∞	45'101	-	
unidentified		414.22761	1	3.6	100 - 23.8		81'700	-	
colchicine	medical alkaloid	400.17547	1	2.8	100 -	NA *	5'064	-	
unidentified		297.12346	1	3.6	100 - 16.7	1'754'131.0	40'751	0.003	
unidentified		414.21841	1	3.5	100 - 3.19	103'697.4	19'364	0.019	
unidentified		410.19585	1	4.1	100 - 22.4	43'304.6	5'248	0.014	
**trihydroxy-methyl-diprenylxanthone**	**food**	**412.21208**	**1**	**3.5**	**100 - 25.5**	**6'145.3**	**687'535**	**12**	**A**
**triamcinolone**	**corticosteroid drug**								
unidentified		430.22259	1	3.3	100 - 25.2 - 0.288	2'292.7	20'299	1	
physalin	food	546.23408	1	2.3	100 - 30.8	2'166.4	152'994	17	
unidentified	Amisulpride : antipsychotic drug	402.20651	1	4.1	100 -	1'648.2	61'968	7	
**drotaverine**	**antispasmodic drug**	**398.23280**	**1**	**3.2**	**100 - 25.9**	**1'463.2**	**2'741'610**	**316**	**B**
unidentified		560.21325	1	2.6	100 - 30.2 - 4.4	1'149.6	209'409	41	
**endoxifen**	**tamoxifen metabolite**	**374.21130**	**1**	**3.4**	**100 - 29**	**895.4**	**6'777'974**	**2'917**	**C**
curcumin II		384.18066	1	3.0	100 - 24.2	400.8	51'615	27	
unidentified		310.19154	1	3.0	100 - 19	226.1	29'722	54	
unidentified		370.20143	1	2.7	100 - 23.2	136.8	39'913	42	
testosterone sulfate / thalicpureine	endogenous / food	386.19639	1	2.5	100 - 24.6	129.4	19'138	30	
unidentified		396.21723	1	3.3	100 - 25.7	127.7	82'924	124	
unidentified		414.22770	1	3.0	100 - 30.4	125.9	605'732	1'580	
endoxifen isotope	endoxifen isotope	376.21822	1	3.4	100 - 15.7	124.4	214'841	1'912	
unidentified		698.34478	1	3.2	100 - 43.3	74.6	36'872	516	
unidentified		588.24449	1	3.4	100 - 30.3 - 2.9	60.6	45'233	139	
phenylbutazone / ergonovine	drug	326.18643	1	3.0	100 - 18.5	57.1	11'084	36	
unidentified		482.12424	1	3.7	100 - 24.1 - 10.2 - 0.544	39.4	86'739	451	
unidentified		372.19611	1	3.3	100 - 68.7	38.7	119'496	1'149	
unidentified		425.30505	1	5.8	100 - 23.5	29.6	10'274	811	
ethyl vanillin isobutyrate	food additive	237.11217	1	3.7	100 - 12.3	26.1	52'572	1'313	
pregnenolone sulfate	pregnenolone metabolite	414.22830	1	3.1	100 - 22.4	25.4	19'337	161	
17-Hydroxypregnenolone sulfate	precursor steroid	430.22259	1	3.7	100 - 18.6	24.1	4'837	33	
unidentified		428.20719	1	3.3	100 - 34.3 - 2.68	23.1	42'014	195	
unidentified		195.10173	1	3.7	100 - 9.65	22.8	118'729	3'921	
unidentified		368.18580	1	2.8	100 - 31.4	20.4	22'362	125	
unidentified		416.23413	1	3.0	100 - 24.2	18.0	20'494	322	
unidentified		364.24810	1	3.5	100 - 18.8	15.0	5'859	91	
PS(14:0/14:0)	phosphatidylserine	702.43572	1	5.4	100 - 30.7	14.1	40'676	1'658	
unidentified		253.10714	1	2.5	100 - 11.8	13.2	37'251	2'492	
hydrojuglone glucoside / coumaroylquinic acid	food	339.10755	1	2.8	100 - 14.6	11.3	4'031	86	
unidentified		441.17552	1	2.8	100 - 22.8	10.8	5'569	105	
unidentified		440.12472	1	2.8	100 - 1.18	10.0	6'417	167	
unidentified		869.68439	2	2.3	52.1 - 100 - 33.6 - 1.06	8.7	74'359	1'534	
unidentified		869.93496	2	2.3	100 - 71.7 - 11.8	8.2	68'072	1'497	
Harmine/ Carbanilide	food, fruits	230.12885	1	0.5	100 - 12.2	6.6	16'935	282	
unidentified		346.33139	1	4.6	100 - 8.37	6.0	9'792	624	
unidentified		400.23910	1	3.2	100 - 47.8 - 9.2	5.2	88'277	1'734	
unidentified		705.62677	2	2.3	69.9 - 100 - 25 - 1.44	3.1	91'686	5'012	

(*): NA : not available; there are no denominators

Table S1
(B)

A B C

1,3,8-Trihydroxy-4-methyl-2,7-diprenylxanthone
SPECTRUM - simulation : C24H26O5 + NH4+:
C24 H30 O5 N1
c(gss, s/p:40)(Val) Chrg 1
R: 50000 Res.Pwr. @FWHM

m/ztheor of A to A+4 isotopes	RIA theor	PQI RIA meas	PQI RIA error	Xcalibur RIA meas	Xcalibur **RIA error**	Xcalibur **MA [ppm]**
412.21185	100			100.0		**0.49**
413.21516	26.86	25.5	5.3%	26.3	**-2.3%**	**0.92**
414.21793	4.49			4.1	**-1.4%**	**0.70**
415.22062	0.56			0.3	**-1.1%**	**1.13**
				internal standard [m/z = 214.08963] =		0.65

Triamcinolone
SPECTRUM - simulation : C21H27FO6 NH4+:
C21 H31 F1 O6 N1
c(gss, s/p:40)(Val) Chrg 1
R: 50000 Res.Pwr. @FWHM

m/ztheor of A to A+4 isotopes	RIA theor	PQI RIA meas	PQI RIA error	Xcalibur RIA meas	Xcalibur **RIA error**	Xcalibur **MA [ppm]**
412.21299	100			100.0		**-2.3**
413.21630	23.67	25.5	-7.2%	26.3	**9.6%**	**-1.8**
414.21886	3.91			4.1	**0.7%**	**-1.5**
415.22149	0.48			0.3	**-0.8%**	**-1.0**
				internal standard [m/z = 214.08963] =		0.7

Dotraverine
SPECTRUM - simulation : C24H31NO4 +H:
C24 H32 N1 O4
c(gss, s/p:40)(Val) Chrg 1
R: 50000 Res.Pwr. @FWHM

m/ztheor of A to A+4 isotopes	RIA theor	PQI RIA meas	PQI RIA error	Xcalibur RIA meas	Xcalibur **RIA error**	Xcalibur **MA [ppm]**
398.23258	100			100.0		**0.3**
399.23590	26.85	25.9	-3.5%	25.6	**-4.7%**	**0.6**
400.23875	4.28			3.7	**-13.3%**	**0.8**
401.24148	0.51			0.4	**-19.9%**	**0.8**
				internal standard [m/z = 214.08963] =		0.6

Endoxifen
SPECTRUM - simulation : C25H27NO2 +H:
C25 H28 N1 O2
c(gss, s/p:40)(Val) Chrg 1
R: 55000 Res.Pwr. @FWHM

m/ztheor of A to A+4 isotopes	RIA theor	PQI RIA meas	PQI RIA error	Xcalibur RIA meas	Xcalibur **RIA error**	Xcalibur **MA [ppm]**
374.21146	100			100.0		**0.2**
375.21476	27.81	29	4.3%	26.8	**-3.6%**	**0.3**
376.21783	4.13			3.9	**-5.5%**	**0.7**
377.22075	0.43			0.3	*-30.2%*	*-1.2*
				internal standard [m/z = 214.08963] =		0.5
